# Transcription factor binding at enhancers: shaping a genomic regulatory landscape in flux

**DOI:** 10.3389/fgene.2012.00195

**Published:** 2012-09-28

**Authors:** Robert-Jan Palstra, Frank Grosveld

**Affiliations:** Department of Cell Biology, Erasmus MC University Medical CenterRotterdam, Netherlands

**Keywords:** enhancer, transcription factor, chromatin looping, transcription, *cis*-regulation

## Abstract

The mammalian genome is packed tightly in the nucleus of the cell. This packing is primarily facilitated by histone proteins and results in an ordered organization of the genome in chromosome territories that can be roughly divided in heterochromatic and euchromatic domains. On top of this organization several distinct gene regulatory elements on the same chromosome or other chromosomes are thought to dynamically communicate via chromatin looping. Advances in genome-wide technologies have revealed the existence of a plethora of these regulatory elements in various eukaryotic genomes. These regulatory elements are defined by particular *in vitro* assays as promoters, enhancers, insulators, and boundary elements. However, recent studies indicate that the *in vivo* distinction between these elements is often less strict. Regulatory elements are bound by a mixture of common and lineage-specific transcription factors which mediate the long-range interactions between these elements. Inappropriate modulation of the binding of these transcription factors can alter the interactions between regulatory elements, which in turn leads to aberrant gene expression with disease as an ultimate consequence. Here we discuss the bi-modal behavior of regulatory elements that act *in cis* (with a focus on enhancers), how their activity is modulated by transcription factor binding and the effect this has on gene regulation.

## INTRODUCTION

Expression of genes is to a large extent directed by regulatory sequences within the promoters of genes. However, early transfection experiments led to the realization that promoters alone were not enough to direct the proper expression of genes. The first enhancers described were SV40 viral repeat sequences that are able to boost expression of a rabbit β-globin construct (Banerji et al., 1981). This enhancement of expression occurred independent of the orientation and location of the enhancer sequence within the reporter construct and this observation became the operational definition of enhancer elements. Soon after the description of viral enhancer sequences the first mammalian enhancer sequences were discovered within the human immunoglobulin heavy Chain locus (Banerji et al., 1983) and it turned out that this enhancer sequence acts in a tissue-specific fashion. Since the first enhancer discovery in humans, many more enhancers have been discovered in different organisms and it is estimated that over 1 million enhancers reside in the human genome (Heintzman et al., 2009). It is also becoming clear that enhancers are marked by the binding of specific chromatin modification factors and the presence of specific histone modifications (Maston et al., 2012). Recent work also suggests that not only protein-coding genes are under the influence of enhancers but that microRNA genes might also be under long-range developmental control (Sheng and Previti, 2011). Although we have come a long way in the 30 years since the first discovery of enhancers, their discovery still remains a challenging task and the mechanism of enhancer action is still largely unknown.

## ENHANCER DISCOVERY BY MAPPING TRANSCRIPTION FACTOR BINDING SITES AND CHROMATIN MODIFICATIONS

Discovery of enhancers has always been a formidable task. DNAseI hypersensitivity mapping was the method of choice since it was observed that regulatory regions within the genome are hypersensitive to DNAseI digestion (Wu, 1980). However, this method was tedious, requiring careful titration of DNAseI concentration, restriction digestion, Southern blotting, and detection with labeled nucleotide probes which yielded only information on particular sequences or loci. The first attempts to identify enhancers on a genome-wide scale did not depend on DNaseI but involved enhancers traps (Hamada, 1986). In this method, a selectable reporter gene driven by an enhancer dependent promoter is randomly integrated in to the genome. Clones in which the reporter gene has integrated within the vicinity of an enhancer can be selected and the enhancer sequences isolated. Subsequent validation of enhancer activity can be done *in vitro* by transiently transfecting luciferase reporter constructs in cell lines or *in vivo* using reporter constructs in transgenic animals. However, this method remains a laborious procedure.

With the emergence of complete sequence information from many different model organisms attempts were made to identify regulatory sequences based on sequence conservation. These bioinformatics attempts were moderately successful (Meireles-Filho and Stark, 2009). However, it has become clear that not all conserved non-coding sequences have a detectable (enhancer) activity and not all enhancers are conserved at the sequence level (Blow et al., 2010; Royo et al., 2011). Recent advances in genome-wide technologies like array technology and more recently high-throughput sequencing are proving to be a game changer for the genome-wide discovery of enhancers. More traditional techniques are currently combined with high-throughput sequencing technologies to identify enhancers on a genome-wide scale and novel approaches of enhancer discovery are introduced. One of the first techniques to be combined with array technology and later high-throughput sequencing as a read out was chromatin immunoprecipitation (ChIP; Barski et al., 2007; Johnson et al., 2007; Mikkelsen et al., 2007; Robertson et al., 2007) and even “old school” DNAseI hypersensitive site mapping has been combined with high-throughput sequencing in order to obtain genome-wide maps of “open” chromatin associated with regulatory regions (Sabo et al., 2006; Hesselberth et al., 2009; Bernstein et al., 2010).

Early genome-wide ChIP experiments found that enhancers are enriched in specific chromatin marks, especially high levels of H3K4me1 in combination with low levels of H3K4me3 appeared to mark enhancer sequences (Heintzman et al., 2007). Later it was found that acetylation of histone H3 at lysine 27 (H3K27Ac) specifically marks active enhancers (Creyghton et al., 2010) and recently it has been reported that in T-lymphocytes di- and tri-methylation of histone H3 at lysine 4 are also correlated with active enhancers (Pekowska et al., 2011). As many more chromatin modifications have recently been identified (Tan et al., 2011), it is to be expected that several of these novel chromatin marks associate with enhancers (Kellner et al., 2012). Transcriptional co-activators like the acetyltransferase and transcriptional co-activator p300 (Visel et al., 2009a; May et al., 2011), the ATAC histone acetyl transferase complex (Krebs et al., 2011) and the ATP-dependent chromatin remodeler CHD7 (Schnetz et al., 2009, 2010) also appear to locate at enhancers. Clusters of tissue-specific transcription factors are hallmarks of enhancers and this fact has been exploited to identify enhancers. He et al. (2011) used a set of five cardiac-specific transcription factors to identify cardiac-specific enhancers that were distinct from p300 bound enhancers. Analysis of the binding of a set of three myogenic-specific transcription factors in combination with p300 binding and enhancer-associated chromatin marks before and after muscle differentiation allowed for the identification of muscle-specific enhancers (McCord et al., 2011). Furthermore, the mysterious highly occupied target (HOT) regions which are bound by many transcription factors but lack their consensus binding motif, function as spatial and temporal enhancers in transgenic assays (Kvon et al., 2012). Conversely, mapping of tissue-restricted enhancers via chromatin marks has lead to the discovery of specific transcription factor binding signatures that correspond to monocyte differentiation states (Pham et al., 2012).

Several laboratories have defined distinct chromatin signatures associated with specific regulatory elements based on the combinatorial analysis of multiple chromatin marks and transcription factor binding patterns (Wang et al., 2008; Ram et al., 2011; Bonn et al., 2012; Cotney et al., 2012; Hoffman et al., 2012), which allows to distinguish between specific enhancer states (Rada-Iglesias et al., 2011; Zentner et al., 2011; Bogdanovic et al., 2012; Cotney et al., 2012). Novel approaches to detect regulatory genomic regions are also emerging like formaldehyde-assisted isolation of regulatory elements (FAIRE) which identifies the more “open” chromatin state associated with enhancers based on differences in phenol extractability of these regions (Giresi et al., 2007). Analysis of different genome-wide data sets is also revealing novel properties of enhancers. Global nuclear run-on followed by high-throughput sequencing (GRO-seq) data revealed that enhancers display bidirectional expression of short transcripts (Melgar et al., 2011; Wang et al., 2011), while an in depth analysis of glucocorticoid receptor (GR)-regulated enhancers revealed that they are enriched in CpG dinucleotides and that their methylation status is cell type-specific and correlate with the accessibility of the enhancers (Wiench et al., 2011).

High-throughput genome-wide approaches have made enhancer discovery a more amendable task. To date, most of these studies have been performed on cell lines but the first attempts to follow enhancer dynamics during development have been successful (Bogdanovic et al., 2012; Cotney et al., 2012). Given the spatial and temporal specificity of enhancers the major challenge for the future will lie in obtaining the proper tissues at the right developmental stage or state of differentiation and performing reliable ChIP-seq on the often limiting amounts of these cells (Bonn et al., 2012).

## TRANSCRIPTION FACTOR-MEDIATED LONG-RANGE ENHANCER–PROMOTER COMMUNICATION

One key feature of eukaryotic enhancers is that they can be located far away from the gene they regulate. How enhancers are able to communicate with their cognate promoters remained a mystery for about two decades. A number of models were proposed which included polymerase tracking, the spreading of chromatin structures, and direct contact between separated elements. The non-contact model (polymerase tracking and chromatin spreading) postulated a role for the intervening chromatin fiber which would propagate a “signal” from the enhancer to the promoter. The contact model, better known as the looping model, proposed that the active enhancer and promoter would reside in close proximity within the nucleus while the intervening chromatin loops out. Although early *in vitro* experiments in prokaryotic systems provided support for the contact model [reviewed in Amouyal (1991)], the first direct *in vivo* evidence in eukaryotes was provided by the phenomenon of transvection in *Drosophila* (Tartof and Henikoff, 1991). The contact model was subsequently experimentally tested by varying the position or distance of genes in a series of experiments using the human β-globin locus (Hanscombe et al., 1991; Dillon et al., 1997).

The subsequent development of new techniques like RNA TRAP (Carter et al., 2002) and chromosome conformation capture (3C; Dekker et al., 2002) and its application to mammalian loci (Tolhuis et al., 2002) allowed the mapping of chromatin folding of gene loci. These studies on the β-globin locus clearly demonstrated that the major regulatory element of the β-globin genes, the locus control region (LCR), resides in close proximity to the genes when active while the intervening chromatin and inactive genes loop out (Carter et al., 2002; Tolhuis et al., 2002). These interactions are developmental stage-specific (Palstra et al., 2003) and dependent on lineage-specific transcription factors (Drissen et al., 2004; Vakoc et al., 2005). Chromatin conformations similar to the ones initially observed within the β-globin locus have been found in several other gene loci in different cell types generally confirming the looping model (de Wit and de Laat, 2012).

3C and its derivatives are currently the method of choice to demonstrate interactions between enhancers and their target genes (de Wit and de Laat, 2012). A major limitation of 3C is the fact that some knowledge of the location of the regulatory elements is needed to design primers. Combining 3C with high-throughput sequencing allows for the unbiased discovery of novel long-range interactions of a specific locus (Soler et al., 2010), especially when combined with ChIP-derived chromatin modifications or transcription factor binding profiles as was demonstrated in a study that identified adipocyte-specific enhancers (Mikkelsen et al., 2010) and a study which identified erythroid-specific enhancers for the *MYB* gene (Stadhouders et al., 2011). One of the remaining drawbacks of this approach is that it still relies on a single locus for a viewpoint and is therefore not truly unbiased. A Chia-PET approach that focuses on either enhancer marks (Chepelev et al., 2012) or promoter-associated RNA polymerase II (RNA pol II; Li et al., 2012) in part circumvents this limitation. A truly unbiased method like Hi-C could in principle detect all long-range enhancer–promoter interactions in a cell population although limitations in sequencing depth and limitations of the bioinformatic tools available currently restricts the resolution of this approach (Lieberman-Aiden et al., 2009). However, taking the fast developments in high-throughput sequencing and bioinformatics analysis into account it may be in the not too distant future that enhancer–promoter interactions are routinely identified using Hi-C. In fact, a first glimpse of tissue-specific promoter–enhancer interactions has been observed in a recent Hi-C study (Dixon et al., 2012).

Binding of lineage-specific transcription factors to enhancers and promoters plays a vital role in the establishment/maintenance of long-range promoter–enhancer interactions. There appears to be a distinct set of transcription factors that tend to bind to promoters and a distinct set that tend to bind at distal regulatory elements (Lan et al., 2012). Analysis of Hi-C and ENCODE data obtained in erythroid leukemia cells indicated that in general factors bound at promoters interact with factors bound at distal sites (Lan et al., 2012). For some transcription factors their role in chromatin looping has been studied in more detail. In a knock-out mouse model of the erythroid-specific transcription factor EKLF, no long-range interactions between the β-globin LCR and β-major gene are observed and the β-globin locus adopts a chromatin conformation reminiscent of the one observed in erythroid progenitor cells (Drissen et al., 2004). Re-introduction of EKLF restores LCR–β-globin interaction and this also occurs in the absence of protein synthesis demonstrating a direct involvement of EKLF in chromatin looping (Drissen et al., 2004). A similar study on the transcription factors GATA-1 and FOG1 has shown that these factors also play a vital role in LCR–β-globin gene interaction (Vakoc et al., 2005). The role of another erythroid transcription factor, the heterodimeric NF-E2 has been more controversial. One study demonstrated that chromatin looping was independent of NF-E2 in a knock-out mouse model of the NF-E2 p45 subunit (Kooren et al., 2007) while an other study demonstrated NF-E2-dependent chromatin looping in a cellular model system upon knock down of the MafK/NF-E2 p18 subunit (Du et al., 2008). Other lineage-specific factors that have been shown to play a role in chromatin looping are GATA3 and STAT6 in the T-cell lineage (Spilianakis and Flavell, 2004) and OCA-B in the B-cell lineage (Ren et al., 2011).

It is doubtful that lineage-specific DNA binding transcription factors are solely responsible for establishing enhancer–promoter interactions. Enhancer bound transcription factors recruit co-activators and general factors of which some have been shown to play a vital role in enhancer–promoter communication. One of the best studied factors is the widely expressed transcriptional cofactor Ldb1. The non-DNA-binding Ldb1 protein is able to interact with multiple transcription factors and mediates interactions between them (Matthews and Visvader, 2003). In erythroid cells, Ldb1 is part of a large complex that contains the core factors TAL1, LMO2, E2A, and GATA1 which is recruited to E boxes and GATA elements in, for example, the β-globin LCR and promoter (Wadman et al., 1997; Soler et al., 2010). Knock-down of Ldb1 in erythroid cells results in an impaired long-range interaction between the β-globin LCR and β-major promoter and a failure to activate β-major expression (Song et al., 2007). A recent report demonstrated that artificial tethering of the self association domain of Ldb1 to the β-globin promoter is able to induce a chromatin loop between the β-globin LCR and promoter and this was sufficient to induce expression of the β-globin gene (Deng et al., 2012b). Other general factors implicated in chromatin loop formation between enhancers and promoters are Brg1, the ATPase component of the SWI/SNF nucleosome remodeling complex (Kim et al., 2009) and the general transcription factor TFII-I (Ren et al., 2011). A different type but very interesting general nuclear factor involved in chromatin looping is cohesin. It is best known for its role in holding together sister chromatids during mitosis, but more recently it has been recognized that cohesin is intimately linked to transcription (Dorsett, 2011; Haering and Jessberger, 2012). The nuclear protein CCCTC-binding factor (CTCF) is thought to partition the genome in separate domains via chromatin loops preventing crosstalk between active and inactive regions (Weth and Renkawitz, 2011; Herold et al., 2012). Recently it was found that these CTCF-mediated chromatin loops are dependent on cohesin (Parelho et al., 2008; Rubio et al., 2008; Wendt et al., 2008; Wendt and Peters, 2009). Interestingly, in murine ES cells cohesin interacts with Mediator and the cohesin loading factor Nipbl and together they participate in chromatin loop formation between enhancers and promoters of ES cell-specific loci (Kagey et al., 2010). Similarly, upon differentiation of mouse erythroid leukemia (MEL) cells cohesin and Nipbl are recruited to the β-globin LCR and β-major promoter coinciding with an increase in transcription. Knock-down of one of these factors resulted in reduced chromatin looping between the β-globin LCR and promoter (Chien et al., 2012). Furthermore, the TBP core promoter associated factor TAF3 cooperates with CTCF and cohesin to mediate long-range chromatin loops between enhancers and promoters in the endoderm lineage (Liu et al., 2011).

**FIGURE 1:**
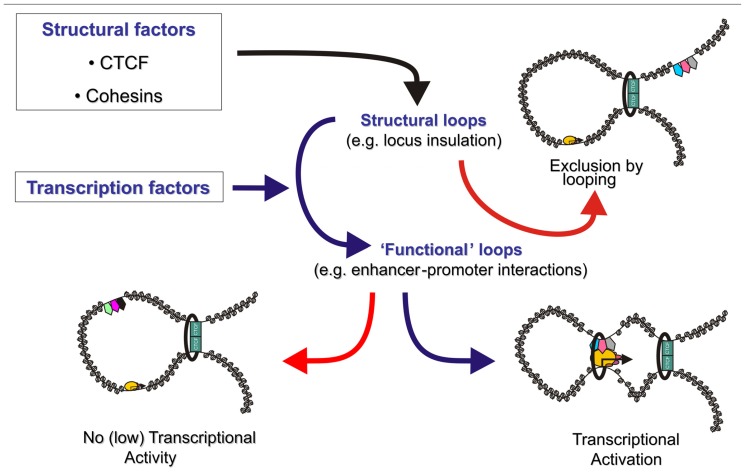
**The genome is organized in transcription factor mediated chromosome loops.** Structural transcription factors like CTCF and cohesin (green rectangles and black ring) co-operate to partition the genome in looped domains. Depending on the location of the anchor points of these looped domains, enhancers are either excluded from a target gene’s domain, effectively blocking activation (top right), or included in the target gene’s topological domain (bottom). Tissue specific and ubiquitous transcription factors (pentagons) either induce enhancer–promoter communication via a chromatin loop (bottom right) or keep enhancers in a silent/poised state (bottom left). Rectangles depict CTCF, the black ring represents the cohesin complex, pentagons depict enhancer bound transcription factors and ovals depict components of the pre-initiation complex. Size of the icons depicts strength of binding or activity.

The general picture that is emerging from these studies is that lineage-specific DNA binding transcription factors bound at promoters and enhancers recruit “looping” factors which setup contacts between distal enhancers and promoters. Such factors appear to form loops within more “structural” loops mediated by general factors like CTCF (**Figure 1**).

The fact that active enhancers reside within close proximity of the active promoters they regulate is currently well recognized. How these interactions are established remains largely unknown. Whether the formation of a chromatin loop is an actively directed process or determined by random collisions has not been elucidated. Several studies suggest that polymerization of nuclear actin might be a driving force in bringing enhancers and promoters together. For example, actin polymerization is necessary for retonic acid induced recruitment of transcription factors to an enhancer element, for the induction of HoxB transcription (Ferrai et al., 2009), for the reactivation of OCT4 during reprogramming by oocytes (Miyamoto et al., 2012), and for the re-localization of gene loci in the interphase nucleus (Chuang et al., 2006; Dundr et al., 2007). Additionally, motor-proteins like nuclear Myosin I and dynein light chain-I have also been reported to be essential for nuclear receptor-induced co-localization of gene loci (Hu et al., 2008). The direct involvement of these factors in establishing enhancer–promoter chromatin loops has however not been shown. Some interpretations of the popular transcription factory hypothesis suggest an alternative actively directed process for bringing enhancers and promoters together (Papantonis and Cook, 2010; Deng et al., 2012a). In this view polymerases bound to enhancers would real-in the chromatin fiber until a promoter is encountered which is subsequently activated (West and Fraser, 2005). However, promoter–enhancer chromatin loops remain when RNA pol II transcription is pharmacologically inhibited, suggesting that such a scenario is unlikely (Mitchell and Fraser, 2008; Palstra et al., 2008).

## ENHANCER MODE OF ACTION

How enhancers actually promote transcription of a gene when in close proximity remains poorly understood. What is clear is that enhancer bound transcription factors recruit co-activators either as part of an enhanceosome or flexible billboards (Alvarez et al., 2003; Arnosti and Kulkarni, 2005). In an enhanceosome a multiprotein complex is assembled at the enhancer and spacing of transcription factor binding sites is crucial for its function (Thanos and Maniatis, 1995). A similar model has been suggested for the multi-enhancer β-globin LCR where the hypersensitive sites of the LCR are thought to form a holo complex (Ellis et al., 1996; Milot et al., 1996). Billboard enhancers are more flexible in their architecture since they consist of separate elements that individually are able to modulate transcription and the additive repressive or activating effects of these elements would determine the transcriptional outcome (Arnosti and Kulkarni, 2005).

Traditionally enhancers are thought to enhance recruitment of RNA pol II and the pre-initiation complex to promoters. It has been suggested that enhancers (or LCRs) function by simply increasing the local concentration of transcription factors, which in turn increases the efficiency of transcription (Palstra et al., 2003). Recent studies have shown that many genes contain stalled polymerases and that the transition from initiation to elongation appears to be a rate limiting step under stringent control (Nechaev and Adelman, 2011). It has therefore been suggested that enhancers play a role in facilitating this transition. Indeed, deletion of the β-globin LCR results in severely reduced phosphorylation of the RNA pol II C-terminal domain (CTD) and transcriptional elongation while pre-initiation complex (PIC) assembly and RNA pol II recruitment to the β-globin promoter was only reduced twofold (Sawado et al., 2003). The erythroid Myb gene enhancers are looped to a conserved CTCF binding site in the first intron of the Myb gene. The p-TEFb component Cdk9 is specifically recruited to the enhancer as part of the Ldb1 complex, and the conserved CTCF site in the intron marks a transition between pausing and elongating polymerases suggesting that enhancers are also essential in regulating transcriptional elongation (Stadhouders et al., 2011). Other results were obtained in a recent study were chromatin looping between the β-globin LCR and β-major gene was induced by tethering of a looping factor (Deng et al., 2012b). Recruitment of RNA pol II to the β-major promoter was restored upon induced chromatin looping while transcriptional elongation remained reduced. The lack of transcriptional elongation is in part explained by the failure to recruit and activate the P-TEFb elongation factor in this system which lacks the crucial erythroid-specific transcription factor GATA1 (Deng et al., 2012b). Together, these studies suggest that enhancers have a function in both PIC and RNA pol II recruitment or stabilization and facilitation of the transition between initiation and elongation.

Alternative mechanisms for enhancer function have also been proposed. Recent genome-wide studies have made clear that RNA pol II is recruited to enhancers (De Santa et al., 2010; Kim et al., 2010; Koch et al., 2011) and that these enhancers are transcribed (Melgar et al., 2011; Wang et al., 2011). A role for these transcripts in enhancer function has been suggested (Orom and Shiekhattar, 2011), however their exact role remains uncertain. Although some non-coding (nc) RNAs seem to behave like classical enhancers in reporter assays (Orom et al., 2010), other observations seem to refute a direct role for the generated ncRNA transcript. The activity of the human growth hormone enhancer is for example dependent on the level of enhancer transcription but not on the structure of its ncRNA (Yoo et al., 2012). Another example where non-coding transcripts are linked to enhancer function is the Kcnq1 imprinted domain (Korostowski et al., 2011). In this case, chromatin loop formation between regulatory elements prevents Kcnq1 promoter silencing by the non-coding Kcnq1ot1 transcript. An attractive but as yet untested possibility is that the ncRNAs are involved in promoting/stabilizing the interaction between the enhancer and its target promoter by RNA binding transcription factors at the enhancer and basic complex transcription factors at the promoter. The observation that RNA pol II complexes are recruited to enhancers has lead to a model in which enhancers are able to transfer RNA pol II to promoters either via direct transfer (Leach et al., 2001) or a tracking mechanism (Zhu et al., 2007). Transfer of polymerases from enhancer sequences to promoter sequences was indeed demonstrated in an *in vitro* assay (Vieira et al., 2004). Convincing *in vivo* data to support this model are however lacking and RNA pol II is still recruited to the β-major gene in the absence of an LCR (Sawado et al., 2003).

Enhancers also seem to play a role in polycomb eviction from developmental promoters containing CpG islands by recruiting the histone H3K27me3 demethylase JMJD3 to the promoter (Taberlay et al., 2011; Vernimmen et al., 2011). In fact, the activity of developmental enhancers itself appears to be kept under tight control by members of the polycomb complex and several other histone methyl transferases (Svotelis et al., 2011; Whyte et al., 2012; Zhu et al., 2012). In breast cancer cells, the poised enhancer of Bcl-2 is marked by H3K27me3. Activation of this enhancer requires the inactivation of the H3K27 methylase EZH2 a member of the polycomb complex and the simultaneous recruitment of the histone H3K27me3 demethylase JMJD3 which is under hormonal control (Svotelis et al., 2011). Several enhancers that have ubiquitous activities when tested in transgenic assays are repressed in non-permissive cells by the presence of flanking regions enriched in H3K9me3 at their endogenous location (Zhu et al., 2012). Cell type-specific recruitment of the H3K9 demethylase Jmjd2d alleviates this repression. Conversely, enhancers responsible for maintaining ES cell identity have to be silenced upon differentiation, which occurs through the recruitment of the H3K4/K9 histone demethylase LSD1 (Whyte et al., 2012).

On the other hand, enhancers that have to become active in a specific lineage are kept in a poised state upon stem cell differentiation via the sequential recruitment of lineage-restricted transcription factors. The transcription factor SOX2 is for example bound at neuron-specific regulatory elements in embryonic stem cells, and is replaced by SOX3 in neuronal progenitor cells and later by SOX11 in terminal differentiated neurons (Bergsland et al., 2011).

It is very well possible that enhancer action goes beyond just one activity and that enhancers perform different tasks sequentially during cellular differentiation. Initially, enhancers will keep gene loci in a transcriptionally competent state by sequential recruitment of progressively more lineage-restricted transcription factors. At a later stage, they will assemble and stabilize a pre-initiation complex at the gene promoter via chromatin looping and finally release paused polymerases through recruitment of elongation factors.

## SPLIT PERSONALITIES OF REGULATORY ELEMENTS

As mentioned before, eukaryotic enhancers were operationally defined in transient transfection assays by the ability to activate a reporter gene irrespective of location and orientation relative to the promoter. This does not necessarily mean that these regulatory elements behave in a similar fashion at their native location in the chromatin context of a cell which is subject to a variety of external signaling cues. The activity of enhancer like elements is regulated in a strict temporal and positional manner within a developing organism. A better approach to test the enhancer like abilities of a DNA sequence is to test it linked to a reporter gene via a transgenic approach. Besides the fact that enhancers can switch between multiple active, poised, and repressed states (Creyghton et al., 2010; Rada-Iglesias et al., 2011; Zentner et al., 2011), new studies indicate that a *cis*-regulatory element can have multiple properties simultaneously.

Depending on the assays used, multiple distinct classes of *cis*-regulatory elements can be recognized (Raab and Kamakaka, 2010). Promoters are bound by transcription factors, provide an assembly point for the RNA pol II holo complex and generally designate a more or less defined directional starting point of transcription. Enhancers recruit transcription factors, they can be transcribed and are able to boost expression from a distally located promoter often in a developmental stage and tissue-restricted manner. The action of enhancers can be counteracted by enhancer blockers when placed between the enhancer and promoter. On the other hand, silencers can suppress transcription from multiple positions relative to enhancers and promoters. Finally, insulators are genetic elements that counteract the spread of heterochromatin.

As discussed above genome-wide studies have demonstrated that many enhancers recruit RNA pol II and are transcribed (De Santa et al., 2010; Kim et al., 2010). Similar observations have been made almost two decades ago for hypersensitive site 2 of the β-globin LCR (Tuan et al., 1992). Most of these enhancer transcripts can be polyadenylated but remain short and are not elongated (Kim et al., 2010). Enhancers that are located intragenic however produce long spliced and polyadenylated transcripts and may therefore function as alternative promoters (Kowalczyk et al., 2012). Promoters of tRNA genes on the other hand have been shown to act as either insulators or enhancer blocking elements in yeast (Simms et al., 2008) and mammalian systems (Raab et al., 2011), which is mediated by binding of the general RNA Pol III transcription factor TFIIIC. In *Drosophila*, RNA pol II promoters containing stalled RNA pol II also act as enhancer blocking elements (Chopra et al., 2009). One model for enhancer blocking function, the decoy model, postulates that enhancer blockers interfere with enhancer–promoter interaction by producing inactive interactions between the enhancer blocking element and the promoter or the enhancer. *Drosophila* enhancer blocking elements indeed appear to form chromatin loops with promoters (Erokhin et al., 2011). Some enhancer blockers can also act as silencers in transient transfection assays suggesting that the distinction between these two elements depends on the assay involved (Petrykowska et al., 2008). Interestingly, it has been reported that the β-globin LCR, which is normally a very strong enhancer in erythroid cells, is able to act as a repressor when placed in the right genomic context (Feng et al., 2005). Specific repressors appear to act on enhancers by interfering with loop formation between enhancers and gene promoters (Chopra et al., 2012). Replacement of an activating loop by a repressive loop has also been observed. When the c-Kit gene is active in immature erythroid cells a GATA2-dependent chromatin loop is present between an upstream enhancer and the promoter (Jing et al., 2008). Upon erythroid maturation, GATA1 replaces GATA2 and the activating enhancer–promoter chromatin loop is replaced by a repressive chromatin loop between the promoter and a downstream silencer-like element. Interestingly, several genetic studies in drosophila have shown that enhancer blockers, when placed in the right context, can enhance enhancer–promoter communication or even act as enhancer elements (Rodin et al., 2007; Maksimenko et al., 2008; Soshnev et al., 2008; Fujioka et al., 2009). These observations indicate that enhancer blockers/silencers function, like enhancers, by means of long-range chromatin interactions. In mammalians, the major protein associated with enhancer blocking function is the 11 zinc-finger transcription factor CTCF (Bell et al., 1999), which is known to mediate long-range chromatin interactions (Splinter et al., 2006). Although CTCF is most famous for its role in enhancer blocking, the protein is also involved in gene activation (Weth and Renkawitz, 2011; Herold et al., 2012). Recent genome-wide analysis of enhancer–promoter interactions have indeed indicated that CTCF is associated with a proportion of enhancers and that CTCF mediates the interaction of these enhancers with their target promoters (Handoko et al., 2011; Li et al., 2012; Taslim et al., 2012).

In summary, it seems that the attempt to impose a strict definition on regulatory elements is much more complicated than expected: enhancers can behave like promoters, promoters can act as enhancer blockers, while enhancer blockers can function as enhancers, all dependent on the genomic context of the regulatory element and the specific set of transcription factors recruited.

## ENHANCER TRANSCRIPTION FACTOR BINDING IN DEVELOPMENT, DISEASE, AND PHENOTYPE DIVERSITY

Tight control of transcription is crucial for the proper development of a multi-cellular organism. Enhancers play a crucial role in ensuring the proper spatio-temporal expression of genes by integrating the action of tissue-specific transcription factors and signaling cues (Buecker and Wysocka, 2012; Ong and Corces, 2012). Given the key role that enhancers play in the proper development of multi-cellular organisms it is of no surprise that disruption of enhancer function is a major contributor to pathological states. In fact, disease driven research has been crucial in the discovery and definition of mammalian enhancers. Investigation of γβ-thalassemia for example led to the discovery and characterization of the “super enhancer”-like β-globin LCR (Grosveld et al., 1987). In Dutch γβ-thalassemia, a large deletion removes 100 kb upstream of the β-globin gene but leaves the β-globin gene itself intact (Kioussis et al., 1983; Wright et al., 1984; Taramelli et al., 1986). The mutant locus is in a closed chromatin state and suffers from position effects. Further analysis of the region deleted in γβ-thalassemia revealed strong erythroid hypersensitive sites upstream of the ε-globin gene (Tuan et al., 1985). Cloning of these hypersensitive sites revealed that they impose position-independent, copy number-dependent high level expression on a β-globin transgene defining the operational properties of a LCR (Grosveld et al., 1987). Many other instances of disease causing enhancer disruptions are currently known (Kleinjan and Lettice, 2008). Translocations can either remove enhancer sequences from a locus (Kioussis et al., 1983) or place ectopic enhancers in the vicinity of onco-genes as is observed in non-Hodgkin’s lymphoma (Hayday et al., 1984). Smaller mutations in regulatory elements are also known to contribute to hereditary disease states. For example, several point mutations as well as insertions within the sonic hedgehog ZRS long-range enhancers cause several forms of preaxial polydactyly (Albuisson et al., 2011; Laurell et al., 2012). The effects of sequence variation in enhancer regions are not always catastrophic and can be quite subtle.

In the past decade, genome-wide association studies (GWAS) have identified many single nucleotide polymorphisms (SNPs) which are statistically associated with phenotypic traits and disease states. The majority of the DNA variants identified in GWAS studies are located in non-coding regions without any known function while only a minority (~30%) potentially disrupt the function of genes (Visel et al., 2009b; 1000 Genomes Project Consortium, 2010). Often linkage with unknown causal (non-synonymous coding) DNA variants within a haplotype block is assumed to explain association of non-coding DNA variants with a given trait. However meta-analysis demonstrated that 40% of the disease associated SNPs including their haplotype blocks exclusively involve non-coding sequence (Visel et al., 2009b) suggesting that these regions have a regulatory function. Moreover, a significant proportion of GWAS SNPs overlap with B, T, and ES cell enhancers (Teng et al., 2011), multiple sclerosis associated regions are located in chromatin regions that are active in B-cells (Disanto et al., 2012) and 80% of the colorectal cancer risk SNPs overlap with colon crypt enhancer marks (Akhtar-Zaidi et al., 2012).

**FIGURE 2:**
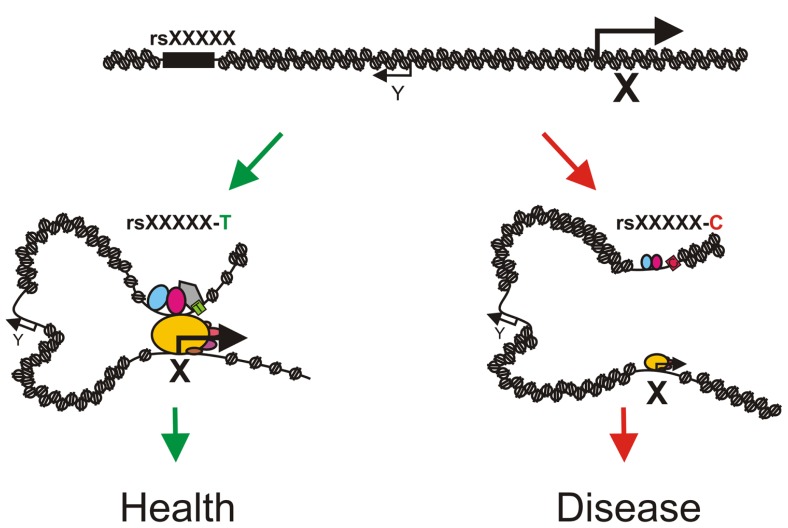
**Model depicting how sequence variation in distal regulatory elements might influence phenotypes or disease states.** A phenotype associated SNP rsXXXXX is located in an enhancer for gene X. The T allele of rsXXXXX binds a transcription factor (the gray pentagon) with high affinity which allows for chromatin loop formation and proper activation of gene X resulting in normal development (left, green arrows). The C allele of rsXXXXX binds the transcription factor with a reduced affinity (light gray pentagon with dashed border) which leads to a less efficient enhancer, absence/reduction of looping and diminished expression of gene X resulting in aberrant or deviated development (right, red arrows). Note that gene Y is located in between the enhancer and gene X and is not regulated by the enhancer. Pentagons depict enhancer bound transcription factors and ovals depict components of the pre-initiation complex. Size of the icons depicts strength of binding or activity.

One can easily imagine that the presence of a SNP might lead to differences in transcription factor binding at regulatory regions which could result in phenotypic changes and even disease (e.g., cancer) due to differences in transcriptional output of the associated genes (**Figure 2**). A study on 10 human lymphoblastic cell lines from different individuals indeed demonstrated that 7.5% of the binding sites for NF-κB and 25% of the RNA pol II binding sites differed between individuals (Kasowski et al., 2010). Differential binding occurred frequently at SNPs and structural variants and was often associated with changes in gene expression. Measurement of the genome-wide allelic imbalance of 24 transcription factors and the transcriptional co-factor p300 indicated that 5% of the binding sites for these factors vary depending on the sequence difference between alleles (Reddy et al., 2012). Chromatin accessibility to DNaseI also depends on genomic variation in lymphoblastoid cell lines and these differences in DNaseI hypersensitivity correlate with differences in transcription factor binding and changes in gene expression (Degner et al., 2012). These observations strongly suggest that many non-coding DNA variants are functional and mark for example enhancers for distally located genes which are involved in the trait under study. Identifying exactly which non-coding SNPs have a regulatory function has been cumbersome, mainly due to the presence of multiple linked non-coding SNPs within a haplotype block, the fact that enhancers are highly tissue- and developmental stage-specific and the lack of proper high-throughput assays to identify enhancer regions. Subsequent identification of the genes regulated by the causative SNPs has proven to be even more difficult, since enhancers and their target genes are often separated by a significant extent of chromatin which can even contain non-target genes. The successful identification of regulatory SNPs and their linked target genes has therefore been limited to few isolated examples.

Several studies on specific risk loci support the notion that in several pathological states SNPs disrupt transcription factor binding sites within enhancers. For example, a risk allele for cleft lip disrupts an AP-2α binding site in an *IRF6* enhancer (Rahimov et al., 2008) and a variant linked to plasma low-density lipoprotein cholesterol and myocardial infarction creates a C/EBPα binding site which results in altered expression of the *SORT1* gene in hepatocytes (Musunuru et al., 2010). Studies on other disease associated loci have demonstrated chromatin loops between the regulatory variant and the genes they regulate. The variant rs6983267 is associated with an increased risk to develop various types of cancers and several studies have demonstrated that this SNP leads to altered TCF7L2 transcription factor binding, altered enhancer activity and that this region loops to the *MYC* proto-oncogene (Pomerantz et al., 2009; Ahmadiyeh et al., 2010; Wright et al., 2010). Similar observations have been made for e.g., variants associated with coronary artery disease (Harismendy et al., 2011), prostate cancer (Zhang et al., 2012), and COPD (Zhou et al., 2012). Not al disruptions of enhancers by SNPs lead to increased disease susceptibility, as they can also have non-pathological effects leading to phenotypic differences. Recently we could demonstrate that rs12913832, a SNP strongly associated with pigmentation in melanocytes, results in differential transcription factor binding at a melanocyte-specific enhancer. This difference in transcription factor binding leads to allele dependent attenuated looping between the enhancer and its target the *OCA2* pigment gene (Visser et al., 2012). Interestingly, allelic differences in enhancer activity are not always reflected in differential enhancer–promoter interactions (Wright et al., 2010), suggesting separate mechanisms for chromatin-loop formation and enhancer activity.

Combining genome-wide ChIP, FAIRE, and 3C high-throughput approaches with data derived from GWAS studies promises to boost the discovery of regulatory SNPs. These kinds of studies are crucial to obtain greater understanding of the impact of sequence variations on human health and disease (Chorley et al., 2008; Hawkins et al., 2010; Ernst et al., 2011) or (part of) the normal variation between individuals. Using these genome-wide approaches it will be possible to shift from just describing statistical associations between variants and traits to studies that actually discover the biology behind disease and phenotype associated non-coding variants.

## CONCLUSIONS AND FUTURE PROSPECTS

Knowledge regarding enhancers and enhancer function has exploded in the past decades. Much of the early insight into enhancer function has been obtained from painstakingly dissecting single model loci. Due to the limited amount of loci investigated, the generality of the occurrence of enhancers and their mode of action remained unclear. With the recent advent of high throughput genome-wide techniques we are now able to address the generality of these early observations. Important insights regarding enhancer–promoter communication, the occurrence of enhancers and enhancer function have been obtained. Surprisingly, the regulatory landscape is far more complex and dynamic as anticipated and it appears that each cell type has thousands of enhancers of which many are cell type-specific. Chromatin looping between regulatory elements is widely observed and appears to be a general principle for long-range enhancer–promoter communication.

However, many challenges remain. Little is known about enhancer dynamics during cellular differentiation, how signaling cascades impact on enhancer function, the role of enhancers in evolution and disease susceptibility and how enhancers actually boost transcription. Further refinement of genome-wide techniques to study enhancer function will help to answer some of these questions. Tracking transcription factor binding and chromatin looping during differentiation will provide unprecedented insights into the dynamics of enhancer action. Although genome-wide approaches are currently in vogue to investigate enhancer function, answers to some of the remaining questions will still require the careful molecular dissection of selected model loci.

Even though progress in technologies has been impressive, several limitations remain. ChIP assays require knowledge regarding the factors involved in the regulation of gene loci and good quality antibodies against these factors are not always available. The genome-wide 3C spin offs currently lack resolution, which hampers the accurate determination of the exact contact points mediating enhancer–gene interactions. Furthermore, these methods all depend on protein–protein and protein–DNA cross-linking using formaldehyde requiring a certain amount of time, setting a limit on the temporal resolution of these methods. Information regarding cell-to-cell variability is still lacking, because the majority of the current methods to study enhancer function involve batch assays on many cells. Therefore, the field would greatly benefit from the development of single cell assays to study enhancer function. The integration of genome-wide data with focused, single locus data and single cell data will undoubtedly provide us with new exciting insights into the mechanisms that shape the genomic regulatory landscape in flux.

## Conflict of Interest Statement

The authors declare that the research was conducted in the absence of any commercial or financial relationships that could be construed as a potential conflict of interest.
